# Exosomes: From Garbage Bins to Promising Therapeutic Targets

**DOI:** 10.3390/ijms18030538

**Published:** 2017-03-02

**Authors:** Mohammed H. Rashed, Emine Bayraktar, Gouda K. Helal, Mohamed F. Abd-Ellah, Paola Amero, Arturo Chavez-Reyes, Cristian Rodriguez-Aguayo

**Affiliations:** 1 Department of Experimental Therapeutics, The University of Texas MD Anderson Cancer Center, Houston, TX 77030, USA; rashedmm1977@gmail.com (M.H.R.); bayraktaremine34@gmail.com (E.B.); PAmero@mdanderson.org (P.A.); 2 Department of Pharmacology and Toxicology, Faculty of Pharmacy, The University of Al-Azhar, Cairo 11754, Egypt; goudahelal@gmail.com (G.K.H.); mohamedabd_ellah@yahoo.com (M.F.A.-E.); 3 Department of Medical Biology, Faculty of Medicine, The University of Gaziantep, Gaziantep 27310, Turkey; 4 Centro de Investigación y Estudios Avanzados del IPN, Unidad Monterrey, Apodaca NL CP 66600, Mexico; achavezr@cinvestav.mx; 5 Center for RNA Interference and Non-Coding RNA, The University of Texas MD Anderson Cancer Center, Houston, TX 77030, USA

**Keywords:** tumor-derived exosomes, non-coding RNAs, intercellular communication, biomarkers, exosome-based therapeutics

## Abstract

Intercellular communication via cell-released vesicles is a very important process for both normal and tumor cells. Cell communication may involve exosomes, small vesicles of endocytic origin that are released by all types of cells and are found in abundance in body fluids, including blood, saliva, urine, and breast milk. Exosomes have been shown to carry lipids, proteins, mRNAs, non-coding RNAs, and even DNA out of cells. They are more than simply molecular garbage bins, however, in that the molecules they carry can be taken up by other cells. Thus, exosomes transfer biological information to neighboring cells and through this cell-to-cell communication are involved not only in physiological functions such as cell-to-cell communication, but also in the pathogenesis of some diseases, including tumors and neurodegenerative conditions. Our increasing understanding of why cells release exosomes and their role in intercellular communication has revealed the very complex and sophisticated contribution of exosomes to health and disease. The aim of this review is to reveal the emerging roles of exosomes in normal and pathological conditions and describe the controversial biological role of exosomes, as it is now understood, in carcinogenesis. We also summarize what is known about exosome biogenesis, composition, functions, and pathways and discuss the potential clinical applications of exosomes, especially as biomarkers and novel therapeutic agents.

## 1. Introduction

Exosomes are membrane-derived nanovesicles of about 30–100 nm released by several types of cells, including mast cells, dendritic cells, B lymphocytes, neurons, adipocytes, endothelial cells, and epithelial cells [1]. Notably, tumor cells have been shown to produce and secrete exosomes in greater numbers than normal cells [2]. Exosomes have been found in numerous body fluids, including blood, amniotic fluid, urine, malignant ascites, cerebrospinal fluid, breast milk, saliva, lymph, and bile, under both healthy and morbid conditions [3,4,5].

Exosomes were first observed three decades ago by Pan and Johnstone while studying the maturation process of reticulocytes into erythrocytes. They noted that vesicles, later named “exosomes”, were shed from cultured monolayer cells and retained the transferrin receptor and many membrane-associated proteins [6,7]. Since it was believed that these vesicles were simply removing unnecessary proteins and other molecules from the releasing cells, exosomes were first thought to function only as cellular garbage disposals [8].

It was not until the mid-1990s that exosomes were shown to have an immunological function [9]. Since then, numerous studies have identified exosomes as a means of intercellular communication that play a role in normal physiological or biologically important processes, such as lactation, inflammation, cell proliferation, immune response, and neuronal function [10,11,12]. They are implicated as well in the pathogenesis of thrombosis, diabetes, and atherosclerosis, and also in the development and progression of diseases such as liver disease, neurodegenerative diseases [13,14,15], and, recently, cancer [16].

## 2. Biogenesis

Although details of the underlying mechanism remain incompletely defined, several processes have recently been shown to have a regulatory role in exosome biogenesis [17].

Exosomes are considered a distinct vesicle population that differs from microvesicles by size. Exosomes are defined as vesicles in the range of 30–100 nm, while microvesicles are defined as vesicles in the range of 100–1000 nm. Despite this clear distinction, however, the terms “exosome” and “microvesicle” have been used interchangeably in many published reports [18].

### 2.1. Formation

**Figure 1:**
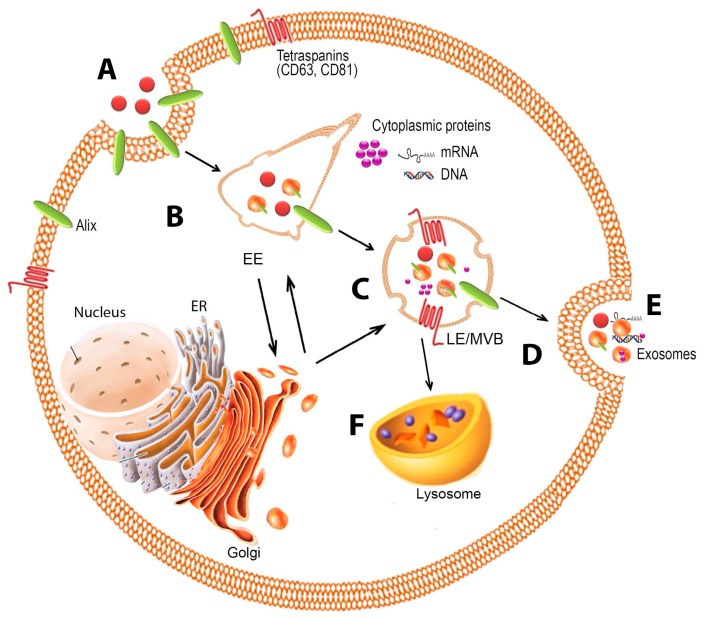
Exosome biogenesis, cargo sorting, and release. Illustration of loading into exosomes of cargo such as nucleic acid and proteins. Endocytosis of the plasma membrane (**A**) results in the uptake of proteins, nucleic acids, and membrane-associated molecules, and formation of the early endosome (EE) (**B**); Upon transformation of the early endosome into the late endosome (LE) (**C**), exosomes are formed by inward budding of the late endosome/multivesicular body (MVB) with the content in a similar orientation as in the plasma membrane (**D**); Fusion of the MVB with the plasma membrane allows for the release of exosomes into the extracellular space (**E**); Alternatively, the MVB may fuse with the lysosome for degradation (**F**). ER: Endoplasmic reticulum.

In general, exosome biogenesis consists of two steps, the inward budding of membranous vesicles of endosomes and their release into a structure known as a multivesicular body (MVB). The formation of MVBs occurs during the maturation of early endosomes into late endosomes with the accumulation of intraluminal vesicles [19]. After maturation, MVBs are directed for fusion with either the lysosome, where their cargo will undergo lysosomal degradation, or the plasma membrane, where their contents will be released into the extracellular space (Figure 1). When MVBs undergo this process, transmembrane proteins are incorporated into the invaginating membrane, maintaining a topological orientation similar to that of the plasma membrane [1,20].

### 2.2. Composition

**Figure 2:**
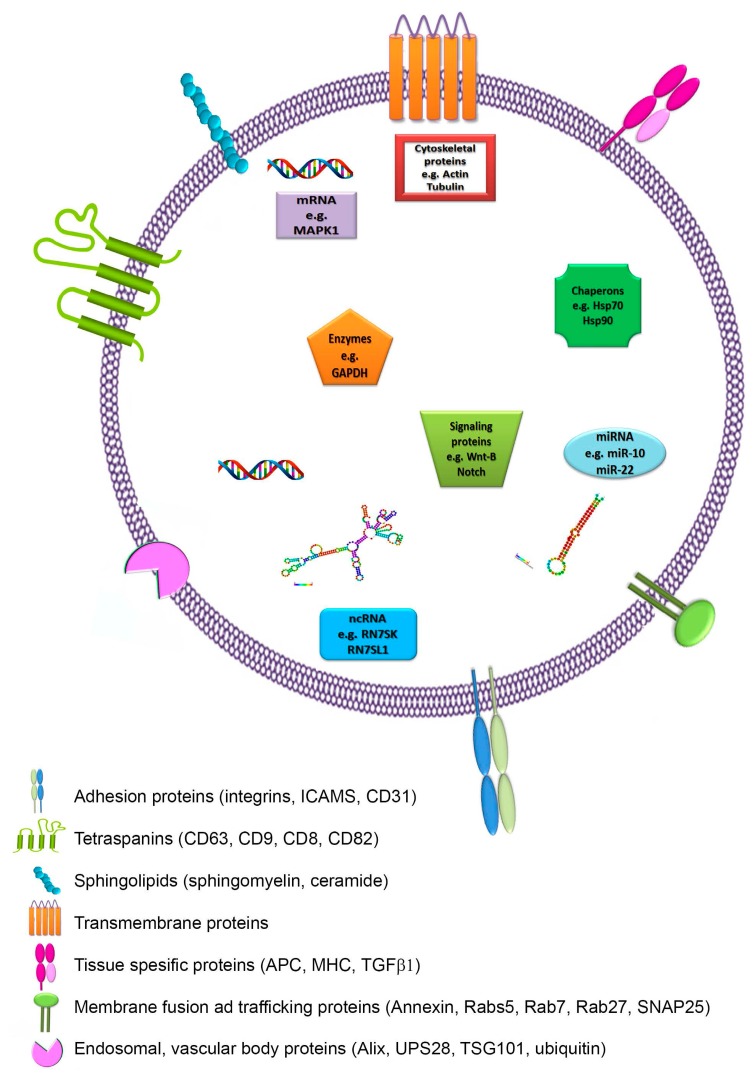
Molecular composition of exosomes. Exosomes are membrane-derived nanovesicles (30–100 nm in diameter) secreted from several cell types. They pack a variety of cellular components, including nucleic acids (e.g., DNA, mRNA, and miRNA), lipids (e.g., cholesterol and ceramide), mRNAs, membrane trafficking proteins (e.g., annexin, Rab 27, SNAP25), chaperones (e.g., Hsp70 and Hsp90), and various tissue-specific proteins involved in antigen presentation as integrins and tetraspanins (CD9, CD63, CD81, and CD82) as well as MHC-I and -II (Major Histocompatibility Complex).

The composition of exosomes differs from cell type to cell type. According to the most recent version of the exosome content database, Exocarta (Version 4), exosomes from various organisms and various cell types have been characterized as containing 4563 proteins, 194 lipids, 1639 mRNAs, and 764 miRNAs [21]. The protein content largely depends on the exosome’s cellular origin and is generally enriched for certain molecules, including targeting and fusion proteins (e.g., tetraspanins, lactadherin, and intergrins), cytoplasmic enzymes (e.g., GAPDH, peroxidases, pyruvate kinases, and lactate dehydrogenase), chaperones (e.g., heat shock proteins Hsp60, Hsp70, Hsp90, and the small HSPs), membrane trafficking proteins (e.g., Rab proteins, ARF GTPases, and annexins), proteins involved in MVB formation (e.g., ALIX, TSG101, and clathrin), cytoskeletal proteins (e.g., actin and tubulin), signal transduction proteins (e.g., protein kinases and heterotrimeric G proteins) (Figure 2) [22].

Exosome-specific protein conformation may be subject to the cell type or tissue birthplace from which it originates and may differ according to the physiological changes and stimulation that the cell underwent. For example, antigen-presenting cell-derived exosomes are enriched in antigen-presenting molecules, including major histocompatibility class (MHC)-I and -II complexes, as well as co-stimulatory molecules [23]. Tumor-derived exosomes usually contain tumor antigens in addition to certain immunosuppressive proteins such as FasL, TRAIL, or TGF-β [24]. Very relevant is the fact that exosomes also contain proteins involved in cell signaling pathways, such as the Notch ligand Δ-like 4 [25], Wnt-β-catenin signaling proteins [26], and some proteins involved in intercellular cell signaling, such as interleukins [27].

The main components of exosomes are lipids. Exosomes are enriched in cholesterol, diglycerides, glycerophospholipids, phospholipids, and sphingolipids or glycosylceramides (including sphingomyelin and ceramide) [28]. Besides these lipids, bioactive lipids, such as prostaglandins and leukotrienes, and enzymes activated in lipid metabolism, such as phospholipase C, are also found in exosomes [28,29]. In this way, exosomes function as lipid carriers, allowing the transport of the bioactive lipids they carry to a recipient cell [30]. Excitingly, exosomal content such as the fatty acid docosahexaenoic acid and lysophosphatidylcholine can enhance dendritic cells’ antigenic capacity [31]. In contrast, the exosomes that contain high levels of prostaglandin PGE2 are involved in tumor immune evasion and the promotion of tumor growth [30].

In addition to proteins and lipids, exosomes also contain functional RNA molecules, including mRNAs and other non-coding RNAs such as miRNAs and lncRNAs [32,33]. These exosomal RNAs, in particular the miRNAs, have been shown to function in the recipient cells [34,35]. Even though the effects of other exosomal loads on receiver cells cannot be ignored, miRNAs are important players in the key functions in this process. Sometimes, exosomal pathways can eliminate tumor-suppressor miRNAs that block metastatic progression [36,37]. Nevertheless, a very large body of evidence in the literature clearly indicates the tumor-promoting role of exosomal miRNAs [38,39,40].

Numerous studies have revealed that the amount of RNA contained in exosomes differs significantly from the amount of RNA present in the parental cell and that the exosomal RNA apparently lacks ribosomal RNA [41,42]. Curiously, however, exosomal RNA content in cancer patients is comparable to that in the original tumor; thus the scientific community has become interested in the potential of the exosomal miRNA profile as a diagnostic tool for cancer [3,43]. Notably, large quantities of miRNAs have been detected in tumors but are not present or are present at very low levels in the exosomes released by the parental cells [44,45]. These results indicate that some miRNAs might be preferentially directed for secretion. However, the processing involved in the selection, packaging, and release of these exosomal miRNAs is not understood, and whether these miRNAs could be used as reliable markers of disease is still under consideration. Collectively, the structure of exosomes is recognized to carry a variety of important proteins, lipids, and genetic materials, and cohesively they interact to guide intercellular communications in healthy and disease states [46].

### 2.3. Cargo Sorting

The mechanisms underlying sorting of proteins and lipids into exosomes are largely unknown, although some of the potential mechanisms that have been suggested involve heteromeric protein complexes (e.g., endosomal sorting complex required for transport (ESCRT)) and also associated proteins such as programmed cell death 6 interacting protein (also recognized as ALIX) and tumor susceptibility gene 101 protein (TSG101) [47,48]. ESCRT proteins, including ESCRT-I, ESCRT-II, and ESCRT-III, are need for cargo selection and the inward budding process (away from the cytoplasm). Some of the components of ESCRT, such as vacuolar protein sorting protein 31 (VPS31), vacuolar protein sorting protein 4B (VPS4B), and TSG101, have been found in endosome-like plasma membrane domains that generate exosomes [49]. Tumor cell exosomes have been shown to contain syndecan, syntenin, and ALIX. Down-modulation of any of these proteins reduced exosomal release, and production of syndecan-, syntenin-, and ALIX-containing exosomes was dependent on the normal functioning of the ESCRT machinery proteins [50].

The secretion of syntenin into exosomes is driven by syndecan, and this process induced heparin sulfate clustering. Overexpression of the enzyme heparanase cleaves the heparan sulfate, causing a noticeable increase in the secretion of exosomes. Moreover, heparanase has also been shown to alter exosome protein composition, which was demonstrated by increase of the levels of syndecan-1, Vascular endothelial growth factor (VEGF), and hepatocyte growth factor (HGF) [51]. The small integral membrane protein of the lysosome/late endosome has been shown to be secreted excessively in exosomes, and its overexpression increases exosome release and exosomal accumulation of ALIX and CD63 [52].

In addition to ESCRT, which recognizes ubiquitylated proteins, other ESCRT-independent mechanisms operate to generate exosomes [53]. These unconventional ESCRT-independent pathways seem to be driven by the presence of certain lipids, such as ceramides and lysobisphosphatidic acid [54,55]. Lipid-metabolizing enzymes, including sphingomyelinase, the enzyme that hydrolyzes sphingomyelin into ceramide, and phospholipase D, which hydrolyzes phosphatidylcholine to generate choline and phosphatidic acid, were shown to regulate exosome secretion [56,57]. Another sphingomyelin metabolite, sphyngosine-1-phosphate (S1P), was shown to play a key role in exosome biogenesis. Silencing of S1P1 receptors impairs the formation of CD63-, CD81-, or flotillin-positive exosomes [58].

Finally, ATP-binding cassette transporter A3, which works like a transporter for phosphatidylcholines, has a role in exosome production [59]. Remarkably, the ESCRT-independent sphingomyelinase pathway produces exosomes enriched in tetraspanins, proteins that contain transmembrane domains that may also be involved in endosomal sorting pathways [60]. Moreover, tetraspanins CD9, CD63, and CD81 are involved in exosome biogenesis and protein loading [61,62]. The signals that control the switch between the two mechanisms remain unknown.

As described, the molecular mechanisms that regulate the loading of proteins into exosomes have been extensively studied. However, the mechanisms by which RNA molecules are sorted into exosomes have remained unknown until recently. It has been shown that specific sequence motifs, such as GGAG present in microRNAs (miRNAs), are involved in this sorting and regulate the localization of miRNA molecules into exosomes [63]. The heterogeneous nuclear ribonucleoprotein A2B1 (hnRNPA2B1) has been shown to bind specifically to exosomal miRNAs through the recognition of GGAG motifs and to control their loading into exosomes. Furthermore, it has been reported that the hnRNPA2B1 loaded into exosomes is sumoylated and that this sumoylation controls the binding of hnRNPA2B1 to miRNAs [64]. In another study, it was found that the RNA-binding protein Y-box protein I (YBX1) binds to miR-223 and is also required for the sorting of this miRNA in the cell-free reaction. Furthermore, YBX1 plays an important role in the secretion of miRNAs in exosomes by HEK293T cells [65]. Kosaka and colleagues have shown that inhibition of sphingomyelinase expression reduced the number of exosomal miRNAs [66]. It has been recognized recently that there is a possible correlation between AGO2, a miRNA-induced silencing complex protein, and exosomal miRNA sorting [67,68]. Silencing of AGO2 has been shown to decrease the abundance of the preferentially exported miRNAs in exosomes [69].

Several reports have shown that exosomes act as transport vesicles for functional long non-coding RNAs (lncRNAs) such as TUC339, ROR, MALAT1, HOTAIR, and GAS5, which may induce cancer-like phenotypes and increase chemoresistance within the recipient cells [33,70,71]. The mechanism for loading lncRNAs into exosomes is currently unknown. Recent observations suggested that specific RNA-binding proteins such as ELAVL1 may play an important role in directing lncRNAs for exosomal transport [72].

### 2.4. Release

The release of exosomes into the extracellular environment requires the transport and docking of MVBs as well as their fusion with the plasma membrane [73]. Many proteins have been implicated in the secretion of exosomes, but the precise mechanism of vesicle release remains elusive and is likely to vary among different cells. It has been proposed that exosome release is a Ca^2+^-dependent [74] and pH-dependent [75] process. 

In some tumor cells, however, exosome release depends on the Rab GTPase family, whose members, such as RAB11, RAB27A, and RAB31, are important regulators of membrane trafficking [76,77,78]. Cells with mutant forms of these proteins release fewer exosomes [79]. Rab family member proteins also have been reported to play a role in exosome secretion, specifically Rab35, which regulates exosome secretion by interacting with the GTPase-activating protein TBC1 domain family member 10A–C [80].

The transcription factor p53 has been shown to be involved in exosome release. Activation of p53 through irradiation resulted in the release of greater numbers of exosomes [81]. A p53-regulated gene, *TSAP6*, was shown to enhance exosome production in cells undergoing a p53 response to stress [82]. Moreover, Lespagnol and colleagues provided direct evidence that exosome production is severely compromised in *TSAP6*-null mice [83].

Other studies have described different mechanisms of exosome secretion that involve the soluble *N*-ethylmaleimide-sensitive factor attachment protein receptor (SNARE) protein YKT6 [26]. Briefly, the cytoskeleton and the contractile machinery of the cell move, attracting the opposing membranes with the assistance of the SNARE complex before pinching off the membrane connection and releasing the vesicle into the extracellular space [84,85]. Other proteins involved in SNARE disassembly are vSNARE VAMP7 and ATPase *N*-ethylmaleimide-sensitive factor, which have been reported to stimulate exocytosis of acetylcholinesterase-containing exosomes in the K562 human leukemia cell line [86].

### 2.5. Uptake

It is still controversial whether exosome uptake is cell type-specific [87] and whether it involves membrane fusion or endocytosis [75,88]. Moreover, exosome uptake may be clathrin-dependent or clathrin-independent [73].

Exosome uptake has been shown to occur via clathrin-mediated endocytosis [89], lipid raft-mediated endocytosis [90], heparin sulfate proteoglycans-dependent endocytosis [91], or phagocytosis [87]. Alternatively, exosomes could be internalized by direct fusion with the plasma membrane [75] or through binding to the surface of a recipient cell through exosomal adhesion molecules phosphatidylserine/lysophosphatidylcholine, and cellular receptors (e.g., LFA1, TIM1, and TIM4) [6].

## 3. Diversity in Exosome Function

There is no doubt that exosomes are involved in many physiological functions and processes, both normal and pathological. Originally, exosomes were described as a mechanism for elimination of excessive proteins or undesirable molecules from the cell [6]. It has been shown that exosomes are secreted to discard membrane proteins, such as transferrin receptors, that have become useless in mature red blood cells. Thus, exosomes were long considered a process whereby cells get rid of undesirable proteins and molecules, making the exosomes a compartment for cellular garbage transport and disposal [6,8]. In the last decade, the exosomes’ role as mediators of cellular communication has emerged, and we now have evidence revealing that exosomes control both normal physiological processes, such as immune response and lactation [10], and the expansion and progression of diseases, such as neurodegenerative diseases [15,92] and especially cancer [16]. Exosomes carry out a diverse range of functions and sometimes have opposing effects on the recipient cells depending on their tissue of origin and molecular content [93,94,95].

Here, we discuss in detail what is known about the functions of exosomes in normal and pathological conditions.

### 3.1. Bioactive Roles of Exosomes in Maintenance of Normal Physiology

Exosomes participate in the maintenance of normal physiology, for example, stem cell maintenance and tissue repair [96]. Exosomes have been implicated as morphogen transporters during development and differentiation. They are released by donor cells and spread through the adjacent tissue at different concentrations, enabling cell–cell communication [97,98]. Importantly, several reports have implicated exosomes in stem cell maintenance and plasticity, indicating that stem cell-derived exosomes have a pivotal role in tissue regeneration following injury [99,100]. Exosomes have also been implicated in cell phenotype modulation and tissue regeneration; for example, exosomes derived from hepatic stem cells can promote hepatocyte regeneration [101]. Exosomes also are involved in converting the hematopoietic stem cell phenotype into a liver cell phenotype [102] and in shifting the bone marrow cell transcriptome toward a lung phenotype in vivo [103].

Exosomes also have a role in tissue homeostasis, as in wound healing. For example, a recent study showed that, after injury, epithelial cells increased the number of exosomes transferring *TGFβ1* mRNA, stimulating fibroblast differentiation through the repair and renewal of tissues subsequent to parenchymal damages [104].

Exosomes display a wide variety of immunomodulatory properties. To sustain strong immunostimulatory activity between mature dendritic cells and B lymphocytes that bind tightly to follicular dendritic cells and whose function is presenting antigen-MHC-II complexes to T lymphocytes, exosome release is necessary to maintain this communication [105]. Furthermore, the effects of immune activation can be mediated by exosome-promoted proliferation and survival of hematopoietic stem cells and activation of natural killer cells [31].

Exosomes have been found to have anti-inflammatory functions. Exosomes released from dendritic cells overexpressing IL-4 or IL-10 suppressed delayed-type hypersensitivity reactions in an MHC-II-dependent manner in a mouse model. These exosomes also suppressed the onset and reduced the severity of collagen-induced arthritis [106,107]. Moreover, FasL on the exosomes was also shown to be important for the suppression of delayed-type hypersensitivity reactions [107]. Dendritic cells treated with IL-4 and IL-10 have shown promise in the treatment of inflammatory and autoimmune diseases [108,109]. Plasma exosomes of mice immunized to a specific antigen were shown to have anti-inflammatory functions in the delayed-type hypersensitivity reaction model similar to those of dendritic cell exosomes, suggesting their relevance in vivo [110]. Exosomes may also have a beneficial role in sepsis, through increased phagocytosis of apoptotic cells [111].

Centrally, in addition to classical synaptic neurotransmission, neurons communicate via the secretion of exosomes and exosome-like vesicles that can contribute to a range of neurobiological functions, including synaptic plasticity [112].

Despite the importance of these findings, a better characterization of exosomes and understanding of their effects are needed if we are to further improve their application in the fields of regenerative medicine and immunotherapy. Most studies in these areas were conducted with non-physiological concentrations of exosomes, whereas in vivo investigations of exosome-induced mechanisms are hampered by the lack of insight into their biogenesis.

### 3.2. Pathological Roles of Exosomes in Spreading of Disease

**Figure 3:**
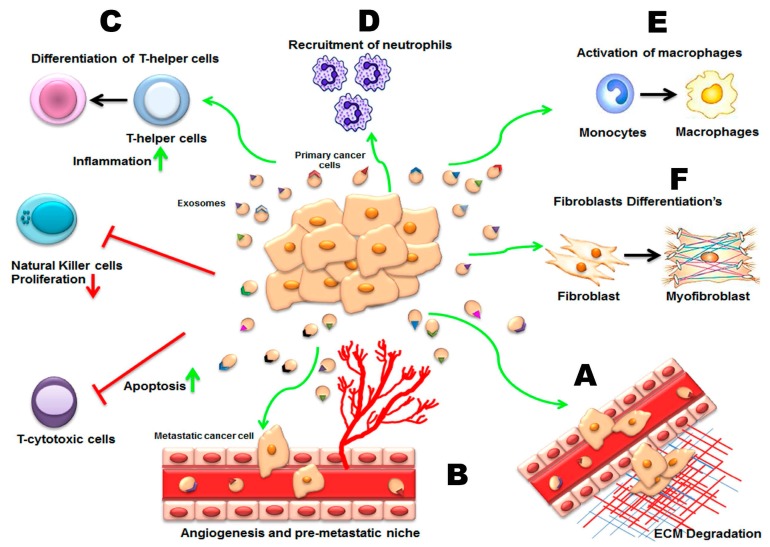
Biological functions of exosomes in tumorigenesis. Exosomes released from tumor cells affect the local tumor microenvironment and are critically involved in tumor initiation, growth, progression, and metastasis by transferring oncogenic proteins and nucleic acids. (**A**) Exosomes travel to distant sites to promote generation of the pre-metastatic niche; (**B**) Angiogenesis is increased and endothelial and stromal cell differentiation is induced, leading to a pro-tumor environment; (**C**) Exosomes have immunosuppressive effects and assist cancers in immune evasion. Cytotoxic T cells are induced to apoptosis, while natural killer cell proliferation is impaired, and T-helper cells differentiate toward a T-regulatory cell phenotype; (**D**) Bone marrow-derived cells are recruited to tumor and pre-tumor tissue where they contribute to cancer development; (**E**) Exosomes are also responsible for the recruitment and activation of tumor-associated macrophages (TAMs) by promoting their polarization. TAMs support diverse phenotypes within the primary tumor, including growth, angiogenesis, and invasion, by secreting a plethora of pro-tumorigenic proteases, cytokines, and growth factors; (**F**) Exosomes can functionally modify fibroblasts by reprogramming these cells to cancer-associated fibroblasts (CAFs), which exhibit myofibroblastic differentiation. Red arrows indicates a negative contribution or repression and green arrows indicate an activation or positive function.

The best understood role of exosomes in disease is their role in tumor biology (Figure 3). One of the hallmarks of cancer cells is that they react with their microenvironment; they can communicate and exchange information by secreted growth factors, cytokines, chemokines, and small molecular mediators (e.g., nucleotides) [113,114].

As very crucial cell-to-cell messenger mediators of communication, exosomes could be notably affecting a recipient cell if they transfer as cargo a specific molecule such as mRNA or non-coding RNA that can alter the gene expression or production of proteins in the recipient cell.

In the following paragraphs, we discuss individually the roles of exosomes in diverse mechanisms, such as metastasis, angiogenesis, hypoxia, and immune escape, which collectively support tumor progression.

#### 3.2.1. Invasion, Metastasis, and Angiogenesis

Because exosomes carry genomic and proteomic materials known to mediate these hallmarks of cancer [32], it has been hypothesized that exosomes secreted by tumor cells have a role in the growth and spread of tumor cells. Indeed, many studies have demonstrated such potential in tumor-derived exosomes. For example, McCready and colleagues demonstrated that Hsp90α-containing exosomes isolated from an invasive cancer cell line could enhance cell migration via activation of plasmin, but the effect was abrogated if an anti-Hsp90 antibody was added to the exosomes [115]. Furthermore, tumor-derived exosomes have been described as having the capacity to establish a pre-metastatic niche with generation of a suitable microenvironment in distant and specific metastatic sites [116,117].

Proteomic analysis of exosomes secreted by human mesothelioma detected the presence of strong angiogenic factors that can increase angiogenesis and vessel density in the neighborhood of tumor cells [118]. In their study on melanoma-derived exosomes, Hood et al. [119] described the pro-angiogenic potential of such nanovesicles, which rapidly stimulated endothelial signaling, important for tissue matrices remodeling and endothelial angiogenesis. The same group reported later that the exosomes of the melanoma cell home to sentinel lymph nodes, which enforces coordinated molecular signals that recruit melanoma cells, inducing extracellular matrix deposition and angiogenesis in the lymph nodes [117]. Consistent with these observations, it has been shown that exosomes from highly metastatic melanoma donor cells augmented the metastatic conduct of primary tumors by continuously “teaching” bone marrow progenitors through the receptor tyrosine kinase MET [120].

One of the major factors that can be involved in the concession of pro-angiogenic activity to tumor exosomes is exemplified by tetraspanins, which are constitutively augmented in exosomes and have been found to contribute to exosome-mediated angiogenesis [121]. The same group that reported that finding later demonstrated that tumor exosomes are directed to non-transformed cells in pre-metastatic niches and organs. This modulates pre-metastatic organ cells, predominantly through transferred miRNA; thus miRNA from a metastasizing tumor arranges or prepares pre-metastatic organ stroma cells for hosting tumor cells [122].

Exosomes derived from a pancreatic tumor cell line overexpressing tetraspanin 8 (Tspan8 or D6.1A) are able to promote tumor growth by their capacity to induce angiogenesis both in vitro and in vivo [123]. Furthermore, exosomal Tspan8 contributes to the selective recruitment of proteins and mRNA into exosomes, including CD106 and CD49d; these two receptors are involved in the binding and internalization of exosomes by endothelial cells. Once the exosomes are internalized, induction of several angiogenesis-related genes, including *VEGF* and *VEGFR2*, is observed in combination with enhanced endothelial cell proliferation and migration and maturation of endothelial cell progenitors [124]. There is also evidence that exosomes from cancerous cells, where the Notch ligand Δ-like 4 was incorporated and where the Δ-like 4 protein was transferred into the cell membrane of host endothelial cells, result in inhibition of Notch signaling and the switch of the endothelial cell phenotype toward tip cells phenotype. The reverted phenotype appears to have a crucial role in vascular development and angiogenesis [25].

#### 3.2.2. Hypoxia

Tumor hypoxia has emerged as a key factor in tumor progression and is associated with poor prognosis and chemoresistance [125]. During hypoxia, exosomes are secreted by tumor cells with increases in angiogenic factors and metastatic potential; this suggests that tumor cells are able to adapt to a hypoxic microenvironment by the secretion of exosomes to promote angiogenesis or facilitate metastasis to a more appropriate microenvironment [126].

There is evidence in several studies involving various cancer models for enhanced exosome release under hypoxic conditions. Borges and colleagues showed in a kidney model that TGF-β1–containing exosomes released by injured epithelial cells can mediate tissue regenerative responses and activation of fibroblasts. These findings strongly suggest the utility of exosome-targeted therapies to control tissue fibrosis [104]. Consistent with this finding was the observation that exosomes derived from hypoxic leukemia cells enhance angiogenic activity in endothelial cells [127]. Similarly, in a highly malignant squamous cell carcinoma model, hypoxic tumor cells modulate their microenvironment and facilitate angiogenesis and metastasis through exosomal secretion of certain proteins [126].

Kucharzewska and colleagues showed in a highly malignant glioblastoma model that exosomes reflect the hypoxic status of glioma cells and mediate hypoxia-dependent activation of vascular cells during tumor development [128]. King and colleagues reported that breast cancer cells grown under hypoxic conditions release more exosomes into their microenvironment via activation of HIF-1α to promote their own survival and invasion [129]. Another study showed that exosomal miR-135b shed from hypoxic multiple myeloma cells enhanced angiogenesis by targeting factor-inhibiting HIF-1 [39].

#### 3.2.3. Cancer Exosomes and Immune Modulation

Tumor-derived exosomes have been reported both to stimulate and to suppress immune response. A significant collection of studies has demonstrated that exosomes can transport antigens such as MHC-I and MHC-II and carcinoembryonic antigen (CEA) from tumor cells to antigen-presenting dendritic cells [130,131,132,133]. The primary dendritic cells, cytotoxic T lymphocytes, induce an immune antitumor response and allow inhibition of tumor growth through MHC-I molecules [130,134]. Similarly, in an ex vivo human model system, dendritic cells pulsed with exosomes derived from malignant effusions proved an effective source of tumor antigens for cross-presentation to CD8^+^ cytotoxic T cells [131]. Moreover, exosomes obtained by stimulating dendritic cells may sensitize adjacent dendritic cells, thereby inducing the immune response [135]. Moreover, surface Hsp70-positive tumor-derived exosomes stimulate natural killer cell activity [136,137]. As result, natural killer cells initiate apoptosis in tumors through granzyme B [136,137].

Despite this, however, an alternative view suggests that exosomes have immunosuppressive effects and assist cancers in immune evasion. For example, tumor-derived exosomes express death ligands such as FasL and TRAIL or high amounts of galectin-9, which can promote T cell apoptosis [138,139,140]. Chalmin and colleagues showed that tumor-derived exosomes activate myeloid-derived suppressor cells and exert TGF-β1–mediated suppressive activity on T cells [141]. In addition, tumor-derived exosomes were shown to promote tumor growth by suppressing natural killer cell function [142,143,144]. Tumor-derived exosomes can also support and expand the immunosuppressive function of regulatory T cells [145,146]. Furthermore, tumor-derived exosomes can block the maturation of dendritic cells and macrophages in vivo and in vitro [147]. Nonetheless, the evidence that exosomes mediate increases or decreases of immunoregulatory functions and proposals that exosomes be administered as immunotherapy must be carefully inspected before translation to further clinical applications.

Growing evidence links tumor metastasis with chronic inflammatory processes and dysregulated activity of various immune cells [148]. Chow and colleagues demonstrate that breast cancer-derived exosomes trigger NF-κB signaling and promote inflammatory cytokine production through Toll-like receptors on macrophages [149]. A similar effect was observed with exosomes derived from malignant ascites of ovarian cancer patients [150]. Another important study showed that miRNAs in cancer-released exosomes can bind as ligands to Toll-like receptors and induce pro-metastatic inflammatory responses [151].

## 4. Exosome-Based Diagnostics and Therapeutics

### 4.1. Exosomes as Therapeutic Target

**Figure 4:**
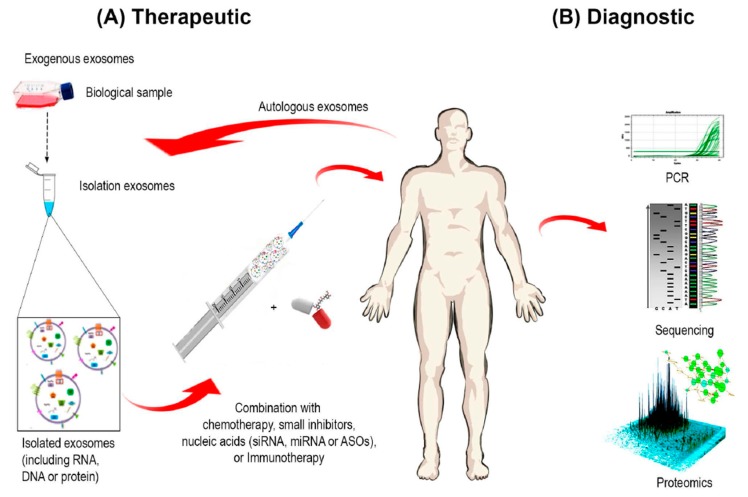
Exosome-based Diagnostics and Therapeutics. Exosomes hold a potential to be used as therapeutic or diagnostics tools. (**A**) Therapeutics: Exogenous or autologous exosomes can be isolated to deliver a desired payload in combination with chemotherapeutics, adjuvants of chemotherapy or as immunotherapy; (**B**) Diagnostics: Biomarkers can be determined to evaluate the expression of proteins, inflammation markers or nc-RNA present in exosomes from biological fluids.

Given that exosome levels are often elevated in correlation with greater severity of different types of cancer [4,152,153], one therapeutic strategy would involve reducing circulating exosomes to normal levels to prevent poor outcomes. With this perspective, many ongoing studies are designed to modulate exosome production either by acting on processes regulating their formation and/or release or by inhibiting their interaction with target cells through specific targeting of their components (Figure 4) [154].

#### 4.1.1. Inhibition of Exosome Formation

Various cellular components are known to be crucial for the formation of exosomes. For example, components of ESCRT are known to be involved in formation of MVBs and intraluminal vesicles [155]. ESCRTs are composed of approximately 30 proteins that assemble into four complexes (ESCRT-0, -I, -II and -III) with associated proteins (VPS4, VTA1, and ALIX) [19]. ESCRT-0, -I, -II, and -III are conserved from yeast to mammals [19]. Several studies have linked the ESCRT-0 protein hepatocyte growth factor-regulated tyrosine kinase substrate (HGS, also known as HRS) to exosome secretion by showing reduced exosome release in HRS-depleted dendritic cells, HEK293 cells [26,156], and tumor cells [157]. Tumor cell exosomes have been shown to contain syndecan, syntenin, and ALIX; overexpression of syntenin induced increases in the ALIX-dependent release of exosomes [50,158].

ESCRT-independent mechanisms of exosome formation have also been described. These mechanisms involve the sphingolipid ceramide or tetraspanins. Small-molecule inhibitors of sphingomyelinase, the enzyme generating ceramide from sphingomyelin, or amiloride attenuate endosomal sorting and exosome production, thereby leading to reduction in tumor growth [56,159].

Furthermore, tetraspanins are enriched in the internal vesicles of MVBs and in exosomes [160]. Expression of tetraspanin Tspan8 could modify both the mRNA content and the protein composition of exosomes secreted by rat pancreatic adenocarcinoma cells [124].

Alternatively, the formation of exosomes may be controlled by specific signaling pathways triggered by Ras homolog family member A [161] or ADP-ribosylation factor 6 [162]. Targeting these pathways may have direct therapeutic significance.

#### 4.1.2. Inhibition of Exosome Release

Several proteins, often including small GTPases of the Rab family, have been implicated in the secretion of exosomes. Rab27a and Rab27b, as well as their effector proteins, are important regulators of exosome release [77,163]. Interestingly, silencing Rab27a by RNA interference disrupted exosome-dependent and -independent mechanisms that modify the tumor microenvironment and can also reduce tumor growth and metastasis [164]. Other Rab proteins such as Rab11 and Rab35 might serve as alternative targets for impairing the release of exosomes by inhibiting the docking of MVBs with the plasma membrane [80,165]. There is also evidence of the involvement of lipids in the regulation of exosome secretion. Downregulation of the diacylglycerol kinase α protein resulted in inhibition of the secretion of Fas ligand-containing exosomes [166].

The final step of exosome release requires the fusion of MVBs with the plasma membrane. This process is mediated by a membrane-bridging SNARE complex machinery that might include VAMP7 [86]. The R-SNARE protein Ykt6 was reported to be involved in the secretion of exosomes carrying the morphogen Wnt from HEK293 cells [26], but further studies are required to confirm details.

Increased intracellular Ca^2+^ stimulates exosomes secretion, and dimethyl amiloride, an inhibitor of voltage-gated Ca^2+^ channels, decreases stimulated exosome release [74,141]. Finally, cellular microenvironmental pH plays an important role in exosome secretion, as its modification using proton pump inhibitors significantly suppressed exosome secretion [167]. Still, further research regarding inhibition of exosome release is missing. Since exosomes are implicated in intercellular communications, and maintaining normal cellular physiology this represent the most important limitations to be used as therapeutic strategy, due to the potential toxicity and other side-effects.

#### 4.1.3. Inhibition of Exosome Uptake

Cells appear to take up exosomes by a variety of endocytic pathways, including clathrin-dependent endocytosis and clathrin-independent pathways such as macropinocytosis and phagocytosis [87,89,168]. It has been shown that treatment of exosomes with proteinase K significantly reduced uptake by ovarian cancer cells. These results indicate that surface proteins on exosomes may serve as receptors for uptake [20]. The uptake of tumor-derived exosomes seems to be mediated by surface phosphatidylserine, which can be blocked with diannexin [169]. Heparan sulfate proteoglycans have been suggested to function as internalizing receptors of cancer cell-derived exosomes. This uptake pathway seems to be important, because treatment with heparin significantly inhibited exosome-mediated stimulation of cancer cell migration [91]. Heparin also inhibited oncogenic *EGFRvIII* mRNA transfer by interfering with the binding of extracellular vesicles into recipient cells [170]. Moreover, exosome internalization could be inhibited by the knockdown of dynamin2, which is necessary for clathrin- and caveolin-dependent endocytosis [87].

Together, these data support the hypothesis that inhibition of exosome biogenesis, release, or uptake mechanisms may have beneficial effects in the treatment of cancer.

### 4.2. Exosome Removal as a Therapeutic Adjuvant in Cancer

Removal of exosomes from the entire circulatory system has been proposed as a novel strategy to treat cancer. Using an affinity plasmapheresis platform, the biotechnology company Aethlon Medical Inc. (San Diego, CA, USA) has established an adjunct therapeutic approach that decreases systemic secretion of HER2-positive exosomes by tumors and inhibits progression of HER2-positive breast tumors [171].

### 4.3. Exosomes as Cancer Immunotherapy

Tumor-derived exosomes carry antigens and have been used as a source of specific stimulus for the immune response against tumors [130]. In contrast, dendritic cell-derived exosomes have the structure necessary to induce very potent antigen-specific immune responses [172]. Previous studies showed that both tumor-derived and dendritic cell-derived exosomes stimulate tumor antigen-specific CD8^+^ cytotoxic T lymphocyte responses; moreover, these exosomes can induce antitumor immunity in experimental animal models and human clinical trials for colorectal, metastatic skin, and non-small cell lung cancers [173,174]. In mice, tumor-derived exosomes can serve as an antigen delivery system and can prevent autologous tumor development in a CD4^+^ and CD8^+^ T cell-dependent manner [130]. In phase I clinical trials, patients with advanced non-small cell lung cancer or metastatic melanoma vaccinated with dendritic cell-derived exosomes exhibited antitumor immune responses and tumor regression [175,176]. Recent evidence has shown that tumor exosome-loaded dendritic cells efficiently produce an antitumor reaction against autologous tumor cells in patients with malignant glioma [177].

Ascites-derived exosomes are believed to be as efficient as those derived from dendritic cells or tumors in sensitizing dendritic cells and prime cytotoxic T lymphocytes, which kill autologous tumor cells in vitro [131]. Furthermore, the exosomes derived from malignant effusions of ovarian cancer patients have been prepared and are ready for clinical trials [178,179]. Moreover, Dai and colleagues found that ascites-derived exosomes from patients with colorectal cancer in combination with granulocyte-macrophage colony-stimulating factor can efficiently induce potent CEA-specific antitumor immunity in patients with advanced colorectal cancer (Figure 4A) [180].

### 4.4. Exosomes as Cancer Diagnostic and Prognostic Markers

Several lines of evidence show that exosomes are present in many biologic body fluids; exosomes might therefore be considered accessible diagnostic biomarkers that hold great potential for detection of many pathological conditions, including cancer [181].

Over the past few years, several studies have addressed the potential use of exosomes as biomarkers in cancer, as their level in circulating blood correlates with prognosis [43,182,183]. Furthermore, miRNAs loaded into exosomes have been suggested as diagnostic and prognostic indicators for ovarian cancer, lung cancer, colon cancer, prostate cancer, and breast cancer [3,184,185,186,187,188]. In addition to miRNAs, exosomal lncRNAs from cancer patients have been defined as novel tumor biomarkers (Figure 4B) [189,190].

### 4.5. Exosomes as a Drug Delivery System

Exosomes offer distinct advantages as gene therapy delivery vectors as they comprise cellular membranes with multiple adhesive proteins on their surface [191]. Furthermore, their small size and flexibility enables them to cross major biological barriers such as the blood–brain barrier [192]. In contrast to established liposome formulations, exosomes are naturally occurring secreted membrane vesicles with lower toxicity; that they are very well tolerated in the body can be inferred from their ubiquitous presence in biological fluids. Their potential utility in drug delivery is implied by their intrinsic homing capacity [193]. For instance, exosomes derived from melanoma home preferentially to sentinel lymph nodes [117], this homing capacity can be used as targeted delivery system for drugs.

Exosomes are naturally adapted for the transport and intracellular delivery of proteins, mRNAs, miRNAs, various non-coding RNAs, mitochondrial DNA, and genomic DNA [32,194,195]. This makes them a valuable tool for the therapeutic delivery of siRNAs [196,197], miRNAs [198,199], and shRNAs [200]. In addition to transfer of interfering RNAs, other types of therapeutic cargo such as lipophilic small molecules can be loaded into these particles. Anti-inflammatory agent curcumin [201,202] and anticancer agents doxorubicin [203] and paclitaxel [204] have been loaded into exosomes or exosome-like vesicles. Exosome-based drug delivery systems may have precedence in the treatment of cancer owing to their endogenous origin, which minimizes their immunogenicity and toxicity. For instance, some studies have shown that the efficacy of doxorubicin loaded into exosomes was greatly enhanced over that delivered by other delivery systems and caused significantly fewer adverse effects on major organ systems, especially the heart, implying that delivery via exosomes might decrease the major downside of this chemotherapeutic drug (Figure 4A) [203,205].

## 5. Conclusions and Future Perspectives

Recent analyses of the composition and biogenesis of exosomes indicate that tumor cells secrete exosomes that can both block tumor growth by promoting antitumor immune responses and induce tumor growth by attenuating antitumor immunity or promoting angiogenesis and/or metastases to distant tissues or organs. The past decade has witnessed a renewed research interest in exosomes, mostly as a result of the demonstration of their immuno-stimulating effects in vivo. The propensity for these controversial effects is contingent upon the type and state of the host cells, the type and state of the recipient cells, and the microenvironment in which these interactions take place. Even though these studies have prompted the clinical application of exosomes, they have not addressed the mechanisms of biogenesis or cargo sorting or established the physiological relevance of the exosome payload. Ongoing advances in the analysis of the formation of multivesicular compartments will probably unravel the mechanisms of exosome generation, which will allow deepening of our understanding of the exact characteristics of exosomes and their functional role in cancer pathogenesis. Uncovering the physiological role of the entirely new mode of cell–cell communication mediated by exosomes may provide us the tools to further improve anticancer therapeutics and cancer diagnostics.
